# Association of the characteristics of the blood metabolome and gut microbiome with the outcome of methotrexate therapy in psoriasis

**DOI:** 10.3389/fimmu.2022.937539

**Published:** 2022-09-07

**Authors:** Qinwei Qiu, Jingwen Deng, Hao Deng, Danni Yao, Yuhong Yan, Shuyan Ye, Xiaoxiao Shang, Yusheng Deng, Lijuan Han, Guangjuan Zheng, Bhaskar Roy, Yang Chen, Ling Han, Runyue Huang, Xiaodong Fang, Chuanjian Lu

**Affiliations:** ^1^ The Second Clinical Medical College, Guangzhou University of Chinese Medicine, Guangzhou, China; ^2^ State Key Laboratory of Dampness Syndrome of Chinese Medicine, The Second Affiliated Hospital of Guangzhou University of Chinese Medicine (Guangdong Provincial Hospital of Chinese Medicine), Guangzhou, China; ^3^ Guangdong-Hong Kong-Macau Joint Lab on Chinese Medicine and Immune Disease Research, Guangzhou University of Chinese Medicine, Guangzhou, China; ^4^ Guangdong Provincial Key Laboratory of Clinical Research on Traditional Chinese Medicine Syndrome, The Second Affiliated Hospital of Guangzhou University of Chinese Medicine, Guangzhou, China; ^5^ Department of Scientific Research, Kangmeihuada GeneTech Co., Ltd (KMHD), Shenzhen, China; ^6^ BGI Genomics, BGI-Shenzhen, Shenzhen, China

**Keywords:** psoriasis, metabolome, microbiome, methotrexate, multi-omic analyses

## Abstract

Metabolic status and gut microecology are implicated in psoriasis. Methotrexate (MTX) is usually the first-line treatment for this disease. However, the relationship between MTX and host metabolic status and the gut microbiota is unclear. This study aimed to characterize the features of blood metabolome and gut microbiome in patients with psoriasis after treatment with MTX. Serum and stool samples were collected from 15 patients with psoriasis. Untargeted liquid chromatography–mass spectrometry and metagenomics sequencing were applied to profile the blood metabolome and gut microbiome, respectively. We found that the response to MTX varied according to metabolomic and metagenomic features at baseline; for example, patients who had high levels of serum nutrient molecular and more enriched gut microbiota had a poor response. After 16 weeks of MTX, we observed a reduction in microbial activity pathways, and patients with a good response showed more microbial activity and less biosynthesis of serum fatty acid. We also found an association between the serum metabolome and the gut microbiome before intervention with MTX. Carbohydrate metabolism, transporter systems, and protein synthesis within microbes were associated with host metabolic clusters of lipids, benzenoids, and organic acids. These findings suggest that the metabolic status of the blood and the gut microbiome is involved in the effectiveness of MTX in psoriasis, and that inhibition of symbiotic intestinal microbiota may be one of the mechanisms of action of MTX. Prospective studies in larger sample sizes are needed to confirm these findings.

## Introduction

Psoriasis is a chronic, immune-mediated skin disease that impacts approximately 125 million people worldwide (1). The pathogenesis of psoriasis is unknown but is thought to be multifactorial and to involve genetic, environmental, and lifestyle-related risk factors. The association of psoriasis with disorders of metabolism is attracting increasing interest. Aberrant metabolism has a major impact on the occurrence, development, efficacy, and prognosis of psoriasis, while the effects of psoriasis can also trigger profound changes in metabolism (2). For example, obesity is considered to be both a risk factor and a trigger as well as a possible consequence of living with psoriasis (3). Moreover, there is evidence that obesity can aggravate existing psoriasis and that weight loss could reduce its severity in overweight patients (4).

Many studies have included metabolomic analyses of various types of samples (peripheral blood, skin lesions, and urine) from patients with psoriasis and described changes in carbohydrates, lipids, amino acids, and nucleotides (5). Given that psoriasis is a systemic disease, metabolomics based on peripheral blood can provide an overall picture of the relationship between psoriasis and metabolic pathways in the body. Consistent with epidemiological research (6), the levels of substances involved in glucose metabolism circulating in peripheral blood, such as α-ketoglutaric acid, lactic acid, aspartic acid, and glutamic acid, have been found to show an increasing trend in patients with psoriasis (7). Other significant changes involve metabolites in urea circulation (e.g., arginine, ornithine, and citrulline) (7, 8), substrates for synthesis of nucleic acids (e.g., hypoxanthine, pseudouridine, inosine, and phosphoric acid) and their products (purines and pyrimidines) (7–11), materials used for keratinization of the epidermis (e.g., proline and glycine) (8, 12), and materials used for synthesis of neurotransmitters and hormones (e.g., L-phenylalanine) (7). Changes in these metabolites reflect the increased energy requirements associated with rapid synthesis of proteins and production of cytokine for hyperproliferation and differentiation of cells. However, higher levels of inflammatory lipids (e.g., lysophosphatidic acid and lysophosphatidylcholine) (13) and peroxides and lower levels of antioxidant markers (e.g., glutathione) are also observed in the plasma of patients with psoriasis, consistent with a microenvironment characterized by inflammation and oxidative stress (5). Targeting metabolites may be helpful in the treatment of psoriasis. For example, treatment with etanercept, an anti-tumor necrosis factor-alpha agent, normalizes most of the psoriasis-associated abnormalities in plasma levels of several amino acids, including arginine, proline, glycine, serine, threonine, alanine, aspartate, and glutamate (8). Recently, an untargeted metabolomics analysis in a psoriasis-like mouse model showed that (R)-salbutamol can alleviate inflammation by regulating the response of Th17/Treg cell in the pathways used for metabolism of glycerophospholipids, sphingolipids, and arachidonic acid (14). This finding may lead to the development of new therapeutic targets for psoriasis.

Integration of metabolomics and the gut microbiome could generate new hypotheses regarding the pathomechanism underlying psoriasis and the pharmacodynamics. The intestinal microbiota is essential to biological processes in the host, including nutrition, metabolism, immunity, and drug efficacy (15). There is emerging evidence that dysbiosis of microbes in the gut contributes to a variety of metabolic, inflammatory, and immune diseases. For example, changes in Bifidobacterium and Akkermansia correspond to changes in specific short-chain fatty acids (SCFAs) that are associated with a lower fat mass, increased insulin sensitivity, and improved gut barrier function (16), while another microbial metabolite known as imidazole propionate contributes directly to development of insulin resistance (17). Many studies have found a significant association between changes in the composition of gut microbes and psoriasis, although there is a high level of heterogeneity in these studies (18).

Methotrexate (MTX) is the first-line treatment for rheumatoid arthritis (RA), juvenile idiopathic arthritis, and psoriasis. Known as a folate antagonist, MTX may also exert a therapeutic effect *via* the extracellular accumulation of adenosine, production of polyamines, cytokine, matrix metalloproteinases, and prostaglandins or inhibition of pro-inflammatory pathways (e.g., those for NF-κB, and JAK/STAT) (19). However, the mechanisms accounting for the differential efficacy of MTX in inflammatory disorders are not well understood. Recently, Teitsma et al. found an association between certain metabolic pathways in serum and achievement of sustained remission of RA after MTX-based treatment (20). Other interesting findings recently found that the gut microbiome can aid in the prediction of MTX efficacy in these patients (21, 22). For example, MTX has off-target effects on the growth, transcription, and metabolic activity of a diverse range of human gut bacteria with downstream consequences for host immunity, and this altered gut microbiota is associated with the response to MTX (21). These observations suggest that the metabolic status and intestinal microecology of the host are important determinants of the response to MTX in patients with RA. Given that the pathogenesis and pharmacological treatment of psoriasis and RA are similar, we hypothesized that the relationship between MTX and the host metabolic status and gut microbiota seen in patients with RA could also be observed in those with psoriasis. The aim of this study was to clarify the effect of MTX on the serum metabolome and gut microbiome in patients with psoriasis.

## Materials and methods

### Study design

**Figure 1:**
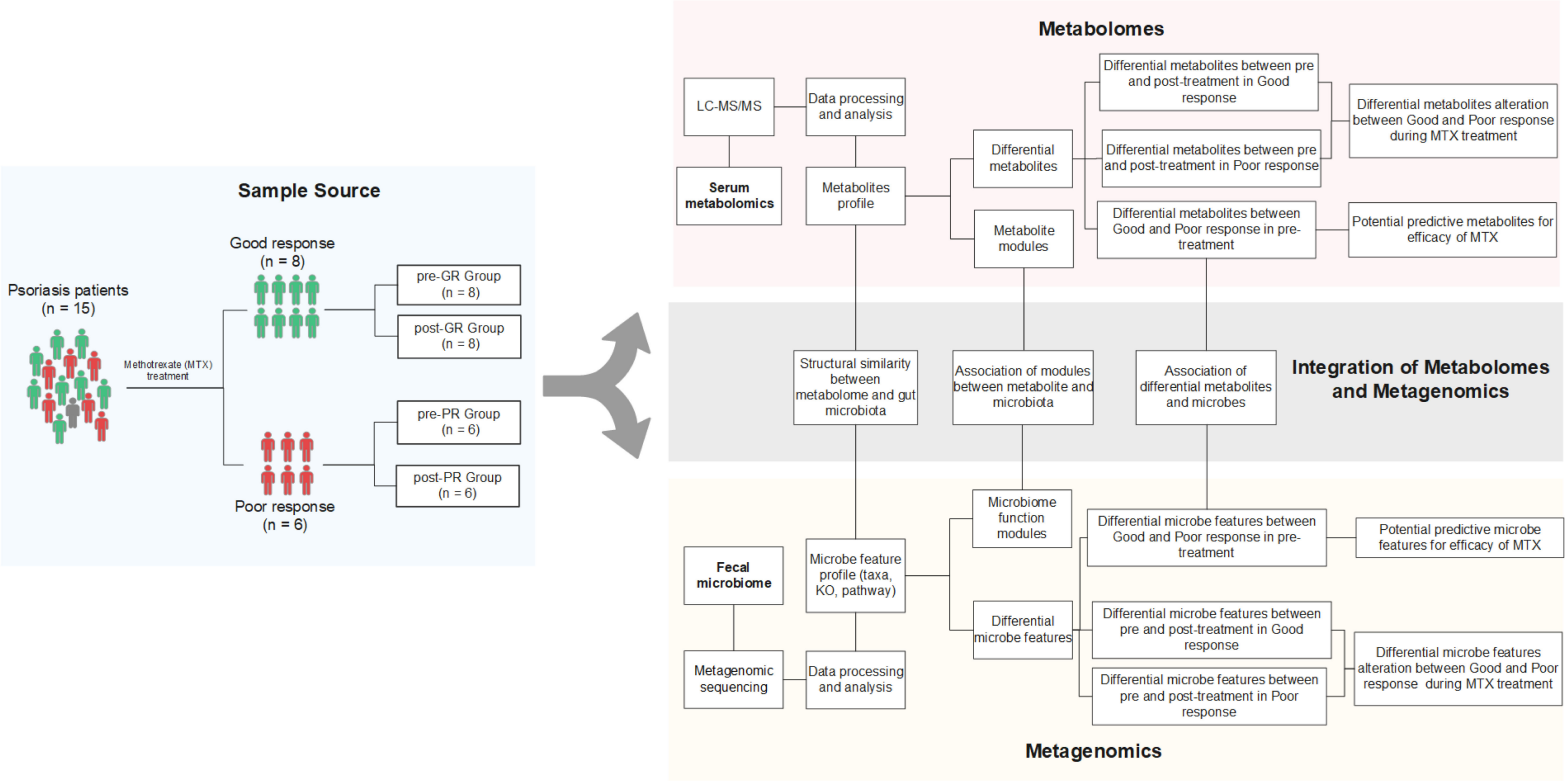
Schematic diagram of the study design. Fifteen patients with psoriasis underwent 16 weeks of MTX therapy and then their clinical improvement was assessed. Eight subjects were assigned to the good response group [at least 75% improvement in PASI (PASI ≥ 75)], while six subjects were assigned to the poor response group [less than 50% improvement (PASI < 50)]. Only one was categorized between these two groups. Fecal samples and blood samples of all 15 patients were collected before and after the treatment. The fecal samples were further investigated by metagenomic sequencing, and the biological information, such as community composition, KO genes, and pathway profiles, was extracted. Metabolites in blood samples were analyzed using an untargeted LC-MS/MS approach. Both omics data were used to screen for the signature associated with differential efficacy. Paired-sample comparison between the pre- and post-treatment was also performed. Further integration of the two omics data occurred on three levels: (i) assessment of the structural similarity between metabolome and microbiota profile (by Procrustes analysis); (ii) correlation analysis between the signatures mentioned above (Spearman rank correlations); and (iii) correlation analysis between microbial function modules and blood metabolite clusters (23).

The study subjects were 15 patients with psoriasis recruited from Guangdong Provincial Hospital of Chinese Medicine in China. To be eligible for inclusion, patients were required to have a clinical diagnosis of psoriasis as defined by the 2018 German S3 guideline for the treatment of psoriasis vulgaris. The study exclusion were as follows (1): pregnancy or lactation (2); serious hematologic, respiratory, or circulatory disease (3); severe infectious disease, tumor, or other malignant disease (4); primary or secondary immunodeficiency or hypersensitivity (5); allergic to the MTX (6); hormonal treatment within the previous 4 weeks, antibiotic treatment within the previous 3 months, or treatment with a biological agent within the previous 6 months; and (7) a cumulative MTX dose of more than 1.5 g already received. If there was a history of treatment with MTX, a washout period of at least 12-weeks was required before inclusion in the study. After enrolment, all study participants received oral MTX for 16 weeks, starting at 7.5 mg/week for 2 weeks and increasing thereafter to 10 mg/week for 14 weeks. At the end of 16 weeks, the patients were divided into a good response (GR) group (at least 75% improvement in the Psoriasis Area and Severity Index [PASI] value [PASI 75]) and a poor response (PR) group (less than 50% improvement in PASI [PASI 50]). Paired stool and peripheral venous blood samples were collected before and after the 16 weeks of intervention. There was one patient with a moderate response (PASI72), whose samples were excluded from the comparison of the GR and PR groups but included in the investigations of putative mechanistic associations between the microbiome, metabolome, and effect of MTX. A flowchart showing the research process is provided in **Figure 1**.

### Metagenomics sequencing and data analyses

Bacterial DNA was isolated from stool samples using a MagPure Stool DNA KF kit B (Magen, China). The extracted DNA was used for PE150 sequencing on the MGI 2000 platform. Reads were filtered from the raw data by SOAPnuke (version 1.5) (24) as follows: removal of reads with >10% of unknown bases; removal of reads with >15-base sequence alignment to adapters; and removal of reads with >40% of low-quality value (*Q* < 20). SOAP2 software (25) was used to exclude potential host contamination. After preprocessing, approximately 10 G of clean data were available for each sample. The taxonomy of the metagenomes was obtained by MetaPhlan2 (26), and the microbial gene and biochemical pathways were characterized by HUMAnN2 (27). The Shannon index, observed species, and Simpson index were used to evaluate for bacterial α-diversity. Differences in microbial characteristics were examined using the Wilcoxon rank-sum test and linear discriminant analysis effect size (LEfSe) (28). Paired tests were used to compare samples obtained before and after treatment.

### Serum metabolomic analysis

Serum metabolites were extracted using previously reported methods (29, 30). Briefly, 100 µl of each serum sample was placed in a 96-well plate, and 300 µl of precooled extract solution (methanol:acetonitrile, 2:1, v/v) was added. After brief vortex mixing, the samples were incubated for 2 h at −20°C and then centrifuged for 20 min at 4,000 rpm. After freeze-drying and redissolving in 150 µl of 50% methanol, the samples were centrifuged for 30 min at 4000 rpm. Supernatants were transferred to autosampler vials for analysis by liquid chromatography with tandem mass spectrometry (LC-MS/MS) analysis. Quality control samples were prepared by mixing aliquots from each sample. We used a high-resolution mass spectrometer (Q Exactive Thermo Fisher Scientific, Waltham, MA, USA) to collect data from both positive and negative ions to improve metabolite coverage. LC-MS/MS raw data processing was performed using Compound Discoverer 3.1 software (Thermo Fisher Scientific) for peak extraction, peak alignment, and compound identification. The data were preprocessed using the following steps (1): probabilistic quotient normalization (31) to obtain the relative peak area (2); correcting the batch effect using quality control-based robust LOESS signal correction (29) (3); calculating the coefficient of variation of the relative peak area in all quality control samples and removal of those with a coefficient greater than 30%; and (4) filtering out of entries without an assigned ID or annotated class according to the Human Metabolome Database (https://hmdb.ca/). Finally, 2,046 compounds (1,590 metabolites in cation mode, 456 metabolites in anion mode) were analyzed. The metabolome profile was dimensionally reduced by principal component analysis (PCA) to examine groups, trends (in intragroup and intergroup similarities and differences), and outliers of the observed variables in the dataset. Hierarchical clustering of the 14 pre-treatment samples was performed using the R package “cluster” v2.1.1 based on Euclidean distance and the ward.D2 clustering method. Based on partial least squares method-discriminant analysis (PLS-DA), differential metabolites were screened according to their variable importance in projection (VIP) value, fold change (FC), and the Student’s *t*-test *p*-value (pre and post group comparison using the paired Student’s *t*-test), with the criterion of simultaneously meeting VIP > 1, |log_2_FC| ≥ 0.25, and *p*-value < 0.05. Functional analysis of these differential metabolites was performed using MetaboAnalyst 5.0 (https://www.metaboanalyst.ca).

### Clustering of co-abundant serum metabolites

Clusters of co-abundant serum metabolites were identified using the R package “WGCNA” v1.70-3 (32). Signed, weighted metabolite co-abundance correlation networks were calculated for all 15 pre-treatment samples. The optimal soft threshold (*β*) was evaluated by the scale-free topology criterion. In brief, for the parameter *β* in the range of 1–30, linear models were established for a node’s connectivity (log i) and the probability of occurrence of the node (log p). When the *R* ^2^ of the fitted linear model exceeded 0.85 for the first time, the corresponding soft threshold of *β* = 9 was chosen for serum metabolite correlations (**Supplementary Figure S6**). Clusters were identified using the dynamic hybrid tree-cutting algorithm with a deepSplit of 2. The minimum number of metabolites constituting a cluster was set to 10.

### Correlation analysis of metabolomics and microbiome

We performed the Procrustes test using the R package “vegan” v2.5-7 to explore the congruence of the two-dimensional shapes produced by PCAs of the two datasets. Spearman correlation analysis was used to examine the association between significant differential bacteria and significant differential metabolites. Functional linkage analysis between gut microbiota KEGG modules, serum metabolites clusters, and the clinical phenotype of psoriasis was performed as described previously (23). In brief, we first selected KEGG modules and metabolite clusters that showed a significant relationship with improvement of the PASI score after treatment (i.e., dPASI). This was done by calculating the Spearman correlation and partial correlation coefficients controlled for body mass index (BMI). The Spearman coefficient between KO genes and metabolite clusters was also obtained for group comparisons (KO genes in vs. not in the module) using the Wilcoxon test. The final coefficient between the KEGG modules and metabolite clusters is presented with the median values subtracted.

### Statistical methods

Categorical data (e.g., sex, alcohol consumption, and smoking status) were tested using the chi-square test and continuous variables using *t*-tests to examine the difference in phenotype index between the groups. The Wilcoxon rank-sum test was used for non-normally distributed data, i.e., metagenomic taxa and pathway. Pretreatment data were compared with post-treatment data using paired Student’s *t*-test or paired Wilcoxon test. In most cases, *p* < 0.05 was set as the threshold for statistical significance. For analysis of the association of the KEGG modules and metabolite clusters, a significant correlation was set to a false discovery rate < 0.1.

### Data availability

The datasets generated for this study can be found in the CNGB Sequence Archive (CNSA) (33) of the China National GeneBank DataBase (CNGBdb) (34) under accession number CNP0002116.

## Results

### Sample characteristics

The characteristics of the study sample are summarized in **Table 1**. There was no significant between-group difference in PASI, body surface area (BSA), or visual analog scale score (VAS) at baseline. However, measures of weight status, such as BMI, body weight, and abdominal circumference, were markedly higher in the PR group. The samples for one patient with PASI72 who had a moderate response were not included in the subsequent group comparisons to maintain statistical reliability.

**Table 1:** Summary of patient demographic and clinical characteristics.

	GR (*n* = 8)	Moderate (*n* = 1)	PR (*n* = 6)	*p*-value (GR vs. PR)
***Demographic characteristics***				
Sex (male/total)	5/8	1/1	5/6	0.58
Age (years)	44 (12.83)	36	36.33 (7.06)	0.18
BMI	21.45 (3.48)	17.63	26.77 (3.58)	0.018 *
Height (cm)	165 (7.48)	165	167.17 (4.17)	0.5
Weight (kg)	58.75 (12.86)	48	74.67 (9.27)	0.02 *
Abdominal circumference (cm)	80.31 (8.94)	82	95.25 (12.78)	0.038 *
***Psoriasis characteristics***				
Duration (years)	12.75 (13.36)	10	14.50 (4.59)	0.74
PASI				
Before	13.49 (3.54)	16.4	13.02 (4.79)	0.84
After	1.29 (1.01)	4.5	8.07 (2.64)	0.00094 ***
BSA				
Before	24.61 (9.60)	39.5	24.58 (7.92)	1
After	0.59 (0.52)	8.8	14.00 (5.26)	0.0015 **
VAS				
Before	6.05 (1.56)	1.9	4.30 (2.18)	0.13
After	1.54 (3.23)	1.2	3.27 (2.48)	0.28
***Lifestyle characteristics***				
Smoking (yes/total)	3/8	1/1	0/6	0.21
Drinking (yes/total)	2/8	1/1	2/6	1

*p < 0.05, ** p < 0.01, *** p < 0.001. Standard deviations are shown in parentheses. BMI, body mass index; BSA, body surface area; GR, good response; PASI, Psoriasis Area and Severity Index; PR, poor response; VAS, visual analog scale score.

### Differences in serum metabolomes at baseline according to efficacy response

**Figure 2:**
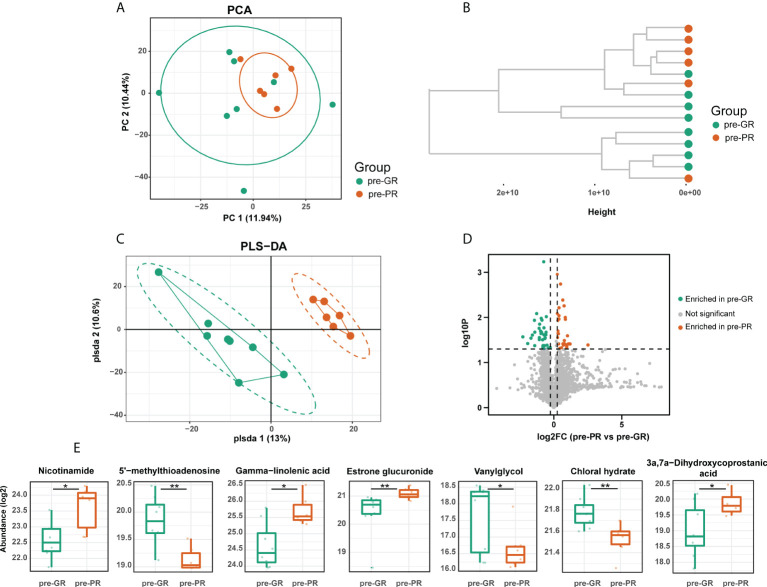
The baseline of serum metabolome comparison between different MTX response groups. **(A)** PCA, **(B)** hierarchical clustering, and **(C)** PLS-DA results describing the dispersing trends of the metabolome profile. **(D)** Volcano plot for differential metabolites between pre-GR and pre-PR. **(E)** Representative differential metabolites involved in the corresponding pathway. Significance is determined by using VIP of PLS-DA > 1, combined with |log2FC| ≥ 0.25 and Student’s *t*-test *p*-value < 0.05. *Student’s *t*-test *p* < 0.05; **Student’s *t*-test *p* < 0.01.

Untargeted serum metabolomics was performed to investigate the metabolic status of the sample in greater detail. First, we compared the baseline metabolite pattern in the 14 individuals (pre-PR vs. pre-GR). Although the pre-GR and pre-PR groups were not separated, the unsupervised PCA showed that samples from the pre-PR group clustered more closely (**Figure 2A**). The results of the hierarchical clustering showed some degree of discrete clusters (**Figure 2B**). Supervised PLS-DA was also performed and revealed good separation between the two groups (**Figure 2C**). Sixty-five metabolites were identified to show a difference in baseline pattern between the pre-GR and pre-PR groups (**Figure 2D**). According to the functional enrichment analysis, these metabolites were mapped onto seven KEGG metabolic pathways, including those for metabolism of nicotinate and nicotinamide, cysteine and methionine metabolism, biosynthesis of unsaturated fatty acids, tyrosine metabolism, primary bile acid biosynthesis, metabolism of xenobiotics by cytochrome P450, and biosynthesis of steroid hormones (**Supplemental Figure S1**). Among the differential metabolites in the corresponding pathway, we noted that an essential nutrient called niacinamide was increased in the pre-PR group, whereas the by-product and allosteric inhibitor of nicotinamide and polyamine synthesis called 5’-methylthioadenosine (35) were abundant in the pre-GR group. This may illustrate the greater nutrient intake or weakened regulation of negative feedback in patients with PR. Another interesting finding was that the pre-GR group had higher levels of chloral hydrate, which interacts with MTX *in vivo* and contributes to slower MTX clearance in children with acute leukemia (36). Surprisingly, the level of gamma-linolenic acid, which is thought to have multiple benefits, including anti-inflammatory effects (35), was found to be significantly higher in the pre-PR group in our datasets. Other differential metabolites mapped to the metabolic pathways including estrone glucuronide, 3a,7a-dihydroxycoprostanic acid, and vanylglycol were abundant in the pre-GR group (**Figure 2E**). In general, there was a discrepancy in the baseline of the metabolome in the different MTX response populations.

### Distinct serum metabolic changes according to the MTX response

**Figure 3:**
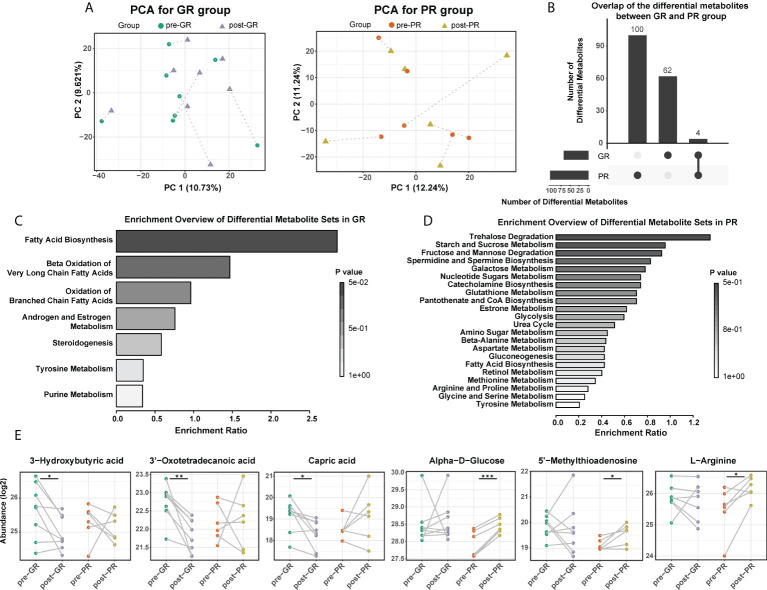
Comparison of serum metabolome before and after MTX treatment. **(A)** PCA plot for the GR and the PR, respectively. **(B)** Overlap of the changed metabolites after MTX treatment in the GR and the PR groups. **(C,D)** Functional enrichment of differential metabolites in the GR **(C)** and the PR group **(D)**, respectively. **(E)** Representative differential metabolites mapped to fatty acid biosynthesis, glucolipid metabolism, and amino acid metabolism. Significance is determined by using VIP of PLS-DA > 1, combined with |log2FC| ≥ 0.25 and the paired Student’s *t*-test *p*-value < 0.05. *Paired Student’s *t*-test *p* < 0.05; **paired Student’s *t*-test *p* < 0.01; ***paired Student’s *t*-test *p* < 0.001.

Next, we analyzed the changes in the metabolome before and after treatment with MTX. PCA results in the GR group showed that nearly all post-treatment (post-GR) samples were shifted along the same direction at the PC1 axis when compared with the pre-treatment (pre-GR) samples; however, no uniform deviation pattern was observed in the PR group (**Figure 3A**). The number of differential metabolites in the PR group was about 50% more than that in the GR group (104 and 66, respectively) but few differential metabolites were shared (**Figure 3B**; **Supplemental Figure S2**). In the GR group, the changed metabolites were mainly mapped onto the pathways for fatty acid metabolism, androgen and estrogen metabolism, steroidogenesis, tyrosine metabolism, and purine metabolism (**Figure 3C**). In particular, levels of many organic and fatty acids (e.g., 3-hydroxybutyric acid, 3-oxotetradecanoic acid, capric acid, 3-oxododecanoic acid, oxalosuccinic acid) decreased significantly after 16 weeks of treatment with MTX, indicating a reduced biological process for biosynthesis of fatty acid (**Figure 3E**). In addition, the activity of other functions like hormone metabolism (17-hydroxyprogesterone, 3, 4-dihydroxymandelic acid) and purine metabolism (uric acid) were also significantly reduced in samples after treatment. For the PR groups, the changed metabolites, including a variety of sugars and amino acids, were involved in more extensive pathways (**Figure 3D**). For example, the level of α-D-Glucose, a fundamental metabolite, was higher in samples obtained after 16 weeks of treatment with MTX, as was the level of L-arginine, a semi-essential amino acid, the metabolism of which is associated with regulation of the immune responses. Interestingly, 5’-methylthioadenosine, which was identified as a differential molecule at the time of the above-mentioned baseline comparison, was also elevated after 16 weeks of treatment (**Figure 3E**). Overall, we found differences in metabolic changes between the GR and PR groups during treatment with MTX that mainly reflected the metabolism of fatty acids, sugars, and amino acids.

### Enrichment of bacteria in fecal microbiota was associated with a poor outcome of MTX treatment

**Figure 4:**
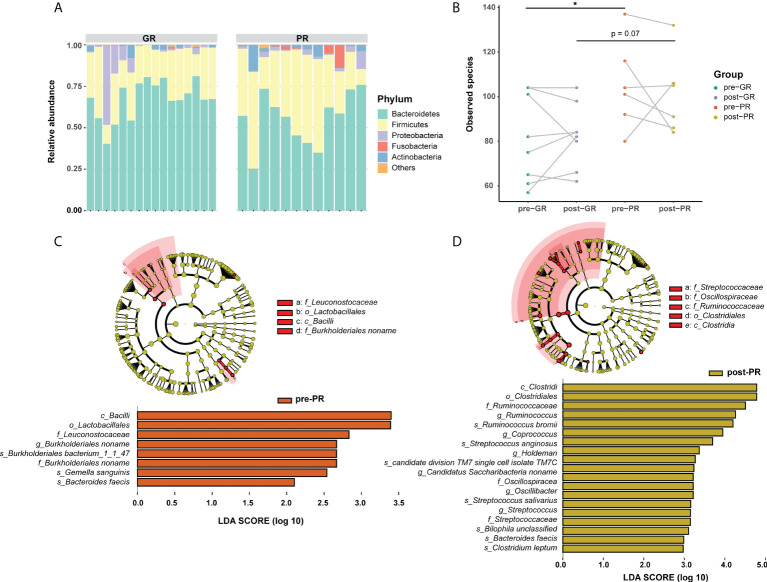
Differences in composition of the gut microbiome between the GR and PR groups. **(A)** Stack plot of phylum composition for each individual. **(B)** Number of observed species comparison for the four groups. *Student’s *t*-test *p* < 0.05. **(C, D)** LEfSe results in baseline samples **(C)** and post-treatment samples **(D)**. Significance is determined by using LDA score ≥ 2.

In view of the interaction between the microbiota and metabolism in the host, we hypothesized that the prognosis in patients with psoriasis who receive MTX may be at least partially associated with the gut microbiome. Although considerable variability in gut microbiota was observed across each sample, the bacterial microbiota was dominated by phylum Bacteroidetes, Firmicutes, Proteobacteria, Fusobacteria, and Actinobacteria in both groups (**Figure 4A**). Bacterial diversity analysis showed a higher richness of species in the PR group than in the GR group (**Figure 4B**; *p* = 0.047 at baseline comparison; *p* = 0.07 after treatment) but no significant difference in the Shannon or Simpson index (Supplemental Figure S3). We then performed LEfSe to identify the discriminatory microbes between the different response groups. Interestingly, all discriminatory microbes were enriched in PR groups. Compared with the GR group, the taxons overrepresented in the PR group were class *Bacilli*, order *Lactobacillales*, family *Leuconostocaceae*, family *Burkholderiales noname*, genus *Burkholderiales noname*, species *Burkholderiales bacterium_1_1_47*, species *Gemella sanguinis*, and species *Bacteroides faeces* at baseline (all LDA scores > 2; **Figure 4C**). After 16 weeks of treatment with MTX, PR enriched more differential bacteria, such as class *Clostridia*, order *Clostridiales*, family *Streptococcaceae*, and family *Ruminococcaceae* (**Figure 4D**). We also performed the same analysis for functional pathways. However, no significant pathway was found at an LDA cutoff > 2. Therefore, we suggest that patients with psoriasis and varying degrees of response have different characteristics in their microbiota, which were reflected in our patients who responded poorly to treatment and more enrichment of features in the microbiota.

### Pronounced alteration of the microbiome before and after MTX treatment in good MTX response group

**Figure 5:**
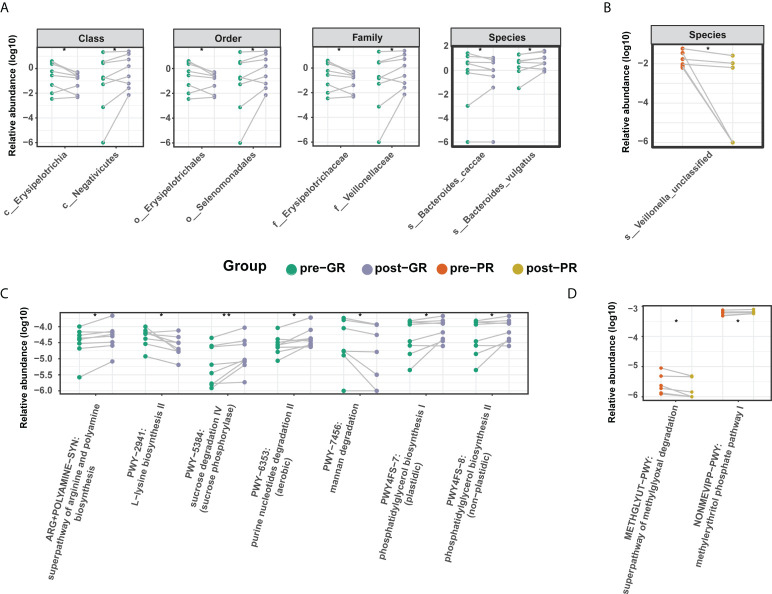
Differential microbiome features before and after MTX treatment. **(A, B)** Differential taxa identified in the GR group **(A)** and PR group **(B)**; **(C, D)** Differential microbial metabolic pathway identified in the GR group **(C)** and PR group **(D)**. Significance is determined by the paired Wilcoxon test. *p < 0.05; **p < 0.01.

To further investigate the effects of MTX on the bacterial microbiota, we analyzed the microbiome before and after treatment with MTX. The diversity in the bacterial community did not change significantly after treatment (**Figure 4A**; **Supplemental Figure S3**). Comparisons of the bacterial profile showed that after treatment with MTX, the relative abundances of phylum Candidatus Saccharibacteria, order Clostridiales, and species *Bifidobacterium longum*, *Bacteroides cellulosilyticus*, and *Streptococcus sanguinis* were decreased, whereas class Negativicutes, family Veillonellaceae, and genus Megamonas were increased in our patients with psoriasis (pair-Wilcoxon rank-sum test *p* < 0.05; Supplemental Figure S4). Regarding the change in microbiota in the GR subgroups, an increased relative abundance of species *Bacteroides vulgatus* and family Veillonellaceae was observed after MTX therapy, while the relative abundance of species *Bacteroides caccae* and family Erysipelotrichaceae were decreased (**Figure 5A**). However, only species *Veillonella unclassified* was significantly changed after 16 weeks of treatment in the PR group (**Figure 5B**).

In terms of microbial metabolic function, the relative abundance of multiple biosynthesis signaling pathways was decreased after treatment, indicating an inhibitory effect of MTX on microbial activity. These pathways included those for several amino acids (such as L-lysine, L-isoleucine, L-threonine, and aspartate), inosine-5’-phosphate, dTDP-rhamnose, and ubiquinol-6. Adenine and adenosine salvage III were also significantly reduced (Supplemental Figure S4). However, there was a difference in the results for the GR and PR groups. In the GR group, levels in the super pathway for biosynthesis of arginine and polyamine, the pathway for degradation of purine nucleotides, and the pathway for biosynthesis of phosphatidylglycerol were increased, while those in the pathway for degradation of mannan were decreased (**Figure 5C**). In the PR group, only two pathways changed after treatment, resulting in an elevated methylerythritol phosphate level and a reduced methylglyoxal degradation level (**Figure 5D**). In general, there was a moderate change in the features of the gut microbiome after treatment with MTX. This change was more pronounced in the GR group than in the PR group.

### Relationship between the gut microbiome, blood metabolome, and outcome of MTX therapy

**Figure 6:**
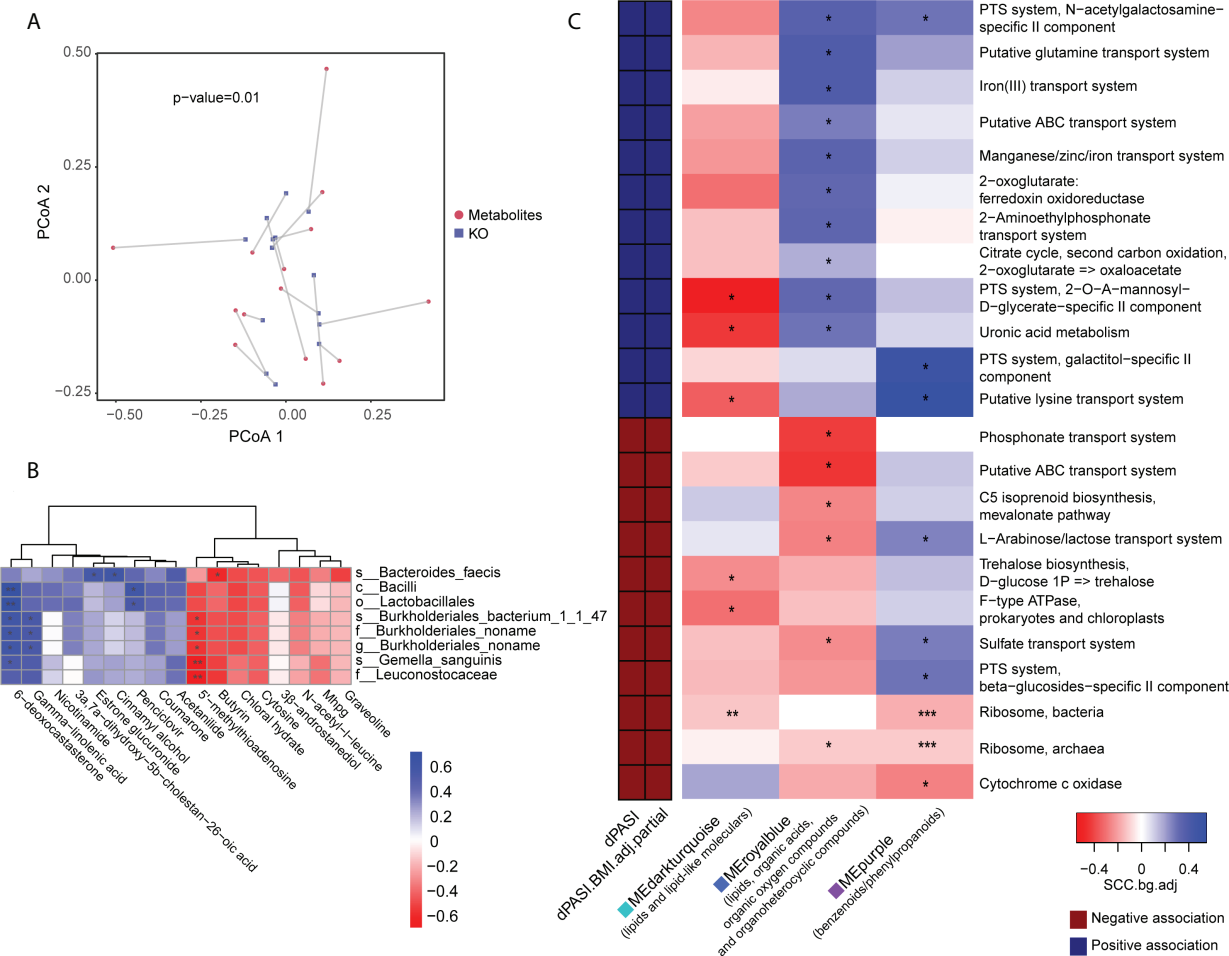
Association of metabolomic and metagenomic data in baseline samples. **(A)** Procrustes analysis for metabolome and microbial KO gene. **(B)** Association of differential bacteria and differential metabolites identified in baseline comparison. **(C)** Association map of the clinical phenotypes, the KEGG function modules of the gut microbiome, and the metabolome modules. The left panel shows significant associations (Wilcoxon rank-sum test *p* < 0.05) between KEGG modules and the dPASI. Red: negative association; blue: positive association. The right panel shows associations between the same KEGG modules and serum metabolite modules (Wilcoxon rank-sum test *p* < 0.05). Coloring represents the median Spearman correlation coefficient between metabolite modules and the indicated KEGG modules, corrected for background distribution [SCCbg.adj; more details in Pedersen et al. (23)]. FDRs are denoted: *FDR < 0.1; **FDR < 0.05; ***FDR < 0.01.

We performed the Procrustes analysis on the microbiome and metabolome. The results at baseline showed a significant correlation between the KO gene and the metabolome (M^2^ = 0.68, *p* = 0.01) (**Figure 6A**) and indicated a structural similarity between the host metabolome and the gut microbiota at baseline that could be disrupted by MTX. We speculated that this association may be linked with the efficacy of MTX. Therefore, subsequent analysis focused on linkages at baseline. Spearman rank correlations between the differential metabolites and microbes revealed that two pre-GR enriched metabolites, namely, 5’-methylthioadenosine and butyrin, had statistically significant negative relationships with pre-PR enriched bacteria, whereas gamma-linolenic acid and 6-deoxocastasterone had positive relationships with those microbes (**Figure 6B**).

We performed data-driven dimensionality reduction for a high-level association study reported by Pedersen et al. (23). Weighted correlation network analysis (WGCNA) yielded 36 modules of closely associated metabolites (**Supplemental Figures 6B, C**), and the module size ranged from 14 to 263. After adjusting for BMI, three modules were significantly related to the rate of reduction (namely dPASI): a “MEdarkturquoise” module enriched in lipids (*r* = -0.63, *p* = 0.016), including glycerolipids, steroid derivatives, and fatty acyls; a “MEpurple” module enriched in benzenoids/phenylpropanoids (*r* = 0.56, *p* = 0.039); and a “MEroyalblue” module without enrichment preferences, including several lipids, organic acids, organic oxygen compounds, and organoheterocyclic compounds (*r* = 0.77, *p* = 0.001). However, many microbial function modules related to dPASI were associated with carbohydrate metabolism (e.g., the citrate cycle, phosphotransferase system, metabolism of uronic acid, and biosynthesis of trehalose), the membrane transport system (e.g., lysine transport, sulfate transport, L-arabinose/lactose transport, and iron transport), and processing of genetic information (ribosome). The modules were then taken forward for cross-domain association analyses (**Figure 6C**). Consistent with the above findings, correlations were found between some of these efficacy-related modules. In particular, the “blue” module that was positively correlated with improvement was also related to most microbial functions with the same direction as dPASI. Another interesting observation was that two microbial ribosome modules were correlated negatively with the dPASI and the three dPASI-associated metabolite clusters, suggesting a potential link between blood metabolism, protein synthesis of gut microbiota, and the response to MTX.

## Discussion

It is known that metabolic abnormalities affect the prognosis of psoriasis (2). In our research, we first noted that the outcomes of treatment with MTX in patients with psoriasis varied according to weight status and then explored the relationship between the blood metabolome and the response to MTX in more detail. The observation that the metabolomes of samples in the PR group clustered more closely indicated a negative schema of metabolites for treatment with MTX. However, when we focused on metabolic changes after MTX, the metabolic profile showed a uniform deviation pattern in subjects in the GR group, which mainly reflected a reduction in fatty acid metabolism. While many metabolites involved in the metabolism of sugar and amino acids were identified as differential factors across treatment, there was a lack of consistency between individuals in the PR groups. Marked elevation of niacinamide has attracted much attention because this metabolite is found in many foods, including meat, fish, milk, eggs, and cereal grains, and can be formed in the body from dietary niacin (37). Higher niacinamide may reflect greater nutrient intake and contribute to overweight in these patients. Another metabolite, 5’-methylthioadenosine, was found to be significantly less in our PR group than in our GR group. It is known that 5’-methylthioadenosine is a by-product of the synthesis of nicotinamide and polyamine, and acts as an allosteric inhibitor in the synthesis pathways for metabolites (35). Previous studies have suggested active synthesis of polyamines in patients with psoriasis, which are converted by monocytes into lymphotoxin products (38). Although inhibition of polyamines has not been considered to be the major mechanism of action of MTX in autoimmune disease, it seems to be helpful for the efficacy of the agent (19). Further evidence is needed to confirm whether lower levels of 5’-methylthioadenosine is an indicator for a weaker suppression in polyamine synthesis and contributing to the poor prognosis of MTX. Metabolites that are abundant in the GR group may also have the advantage of improving the pharmacokinetic properties of MTX. There has been a report (36) of a drug interaction between MTX and chloral hydrate in a patient being treated for acute leukemia. When co-administered with chloral hydrate, the clearance of MTX became markedly slower and the exposure to MTX was significantly increased. Overall, our results suggest a link between the outcome of treatment with MTX and the host’s metabolic status.

Dysbiosis of the gut microbiome is found in many diseases, including psoriasis, and often involves a decrease in alpha diversity (39–42). Interestingly, in our study, microbial diversity was higher, and bacteria were more enriched in the PR group than in the GR group. This finding is similar to that in a recent study by Artacho et al., who reported that patients with RA who responded to MTX had significantly lower microbial diversity in the gut (22). Higher diversity often means a more stable microflora ecosystem. However, in terms of the microecology in disease, the transition from a dysbiotic state to a healthy state requires external forces that are stronger than the stability properties of the system (43). Therefore, higher diversity in the gut microbiota community may hamper the efficacy of therapy. MTX has effects on the growth, transcription, and metabolic activity of a diverse human gut bacteria (21). We also found that a variety of metabolic pathways of gut microbes were reduced after treatment with MTX. Considering that the ability of MTX to interfere with DNA synthesis is widespread across species, we hypothesized that MTX would inhibit the biological activity of intestinal microorganisms in our subjects. Our PR groups showed higher microbial diversity and fewer changes after treatment, suggesting a certain degree of resistance to the effects of MTX.

As mentioned in many studies (15, 44–46), host metabolic status is strongly interconnected with the gut microbiota. By integration of the two omics in our research, we found a broad link between blood metabolites and gut microbial function. Functional modules related to the basic physiological activities of microbes, including carbohydrate metabolism, the transport system, and the ribosome, were also associated with the degree of improvement in the PASI after 16 weeks of treatment. For example, the ribosome lies at the core of bacterial growth, with protein synthesis consuming more than 60% of the cell energy budget (47). In the present study, two ribosome modules were related to the host metabolic clusters containing benzenoids/phenylpropanoids, fatty acyls, and glycerolipids and were inversely correlated with subsequent improvement in disease severity. Our observation of lower levels of biosynthesis of fatty acids in individuals with GR combined with the inhibitory effect of MTX on microbial activity suggests that inhibition of intestinal microbes is a putative mechanism for the efficacy of MTX in psoriasis.

This study had several limitations that will need to be addressed in future work. First, the small sample size decreased the power of the statistical analysis. We are continuing to recruit patients and anticipate that a larger sample size would confirm our present results. Second, more evidence of the relationship between the metabolism of the host and the gut microbiome is required to fill the knowledge gap. The metabolites secreted by bacteria are the main mediators of communication with the host and include SCFAs, branched chain amino acids (BCAAs), trimethylamine N-oxide (TMAO), tryptophan, and indole derivatives (45). The association identified in this research requires validation by further studies combined with microbial metabolomic data and animal experiments. Third, we investigate the fecal microorganisms that are representative of those found in the human large intestine. The pharmacokinetics of MTX are such that only a small amount of the drug pass through the large intestine (19). Animal experiments are required for a more comprehensive examination of the effect of MTX on gut microbes, given that the small intestine is the most appropriate site for sampling the gut microbiome in the human but is difficult to access in clinical practice. Whether it is feasible to regulate the gut microbes by diet or whether conventional medication is needed to modify the therapeutic effect of MTX requires further exploration.

Overall, this study offers new insights into the relationship between the response to MTX treatment, the serum metabolome, and the gut microbiome in psoriasis. Its findings could contribute to a better understanding of the mechanism by which MTX improves psoriasis.

## Data availability statement

Metagenomics and metabolome data that support the findings of this study are available in China National GeneBank Sequence Archive (CNSA) with accession number CNP0002116.

## Ethics statement

The studies involving human participants were reviewed and approved by the ethical committee of the Guangdong Provincial Hospital of Traditional Chinese Medicine (GPHCM B2017-031-02). The patients/participants provided their written informed consent to participate in this study.

## Author contributions

CL, XF, and RH designed the study and supervised the entire investigation. CL, HD, DY, YY, and SY contributed to patient recruitment and sample collection. QQ, JD, YD, and XS performed the bioinformatic analysis. QQ, JD, LinH, HD, GZ, RH, and YC contributed to the primary interpretation of analytical outcomes. QQ wrote the first draft of the manuscript. JD and HD wrote sections of the manuscript. All authors contributed to manuscript revision, read, and approved the submitted version.

## Funding

This project was supported by grants from the following sources: the National Natural Science Foundation of China (No. U20A20397, No. 81873302, and No. 81704081), which had a role in the data collection and patient recruitment; the Science and Technology Planning Project of Guangdong Province (No. 2017B030314166 and No. 2020B1111100005), which had a role in trial design, data collection, and patient recruitment; Science and Technology Planning Project of Guangzhou (No. 202206080006), which had a role in trial design , patient recruitment and data analysis; Innovation Team and Talents Cultivation Program of National Administration of Traditional Chinese Medicine (No. ZYYCXTD-C-202204), which had a role in data collection; Guangdong Provincial Clinical Research Center for Chinese Medicine Dermatology (No. 2020B1111170012), which had a role in patient recruitment, data analysis, and interpretation; the 2020 Guangdong Provincial Science and Technology Innovation Strategy Special Fund (Guangdong-Hong Kong-Macau Joint Lab; No. 2020B1212030006), which had a role in patient recruitment, data analysis, and interpretation; and the Specific Fund of State Key Laboratory of Dampness Syndrome of Chinese Medicine (No. SZ2020ZZ27), which had a role in data analysis and interpretation.

## Acknowledgments

This work was supported by China National GeneBank (CNGB). The authors thank Liwen Bianji (Edanz) (www.liwenbianji.cn) for editing the English text of a draft of this manuscript.

## Conflict of interest

Author LH was employed by Kangmeihuada GeneTech Co., Ltd. Authors BR and XF were employed by BGI-Shenzhen.

The remaining authors declare that the research was conducted in the absence of any commercial or financial relationships that could be construed as a potential conflict of interest.

## Publisher’s note

All claims expressed in this article are solely those of the authors and do not necessarily represent those of their affiliated organizations, or those of the publisher, the editors and the reviewers. Any product that may be evaluated in this article, or claim that may be made by its manufacturer, is not guaranteed or endorsed by the publisher.

## Abbreviations

BS, Body surface area; BMI, Body mass index; FC, Fold change; GR, Good response; LEfSe, Linear discriminant analysis effect size; MTX, Methotrexate; PASI, Psoriasis area and severity index; PCA, Principal component analysis; PLS-DA, Partial least squares-discriminant analysis; PR, Poor response; QC, Quality control; RA, Rheumatoid arthritis; SCFAs, Short-chain fatty acids; VAS, Visual analogue scale; VIP, Variable importance in projection; WGCNA, Weighted gene co-expression network analysis.
