# Supplementary material for: Association of the characteristics of the blood metabolome and gut microbiome with the outcome of methotrexate therapy in psoriasis
Source: Front Immunol. 2022 Sep 7;13:937539. doi: 10.3389/fimmu.2022.937539 (PMC9491226; doi:10.3389/fimmu.2022.937539)
Supplement: Supplementary file 1 [file DataSheet_1.docx]

Supplementary Material

**
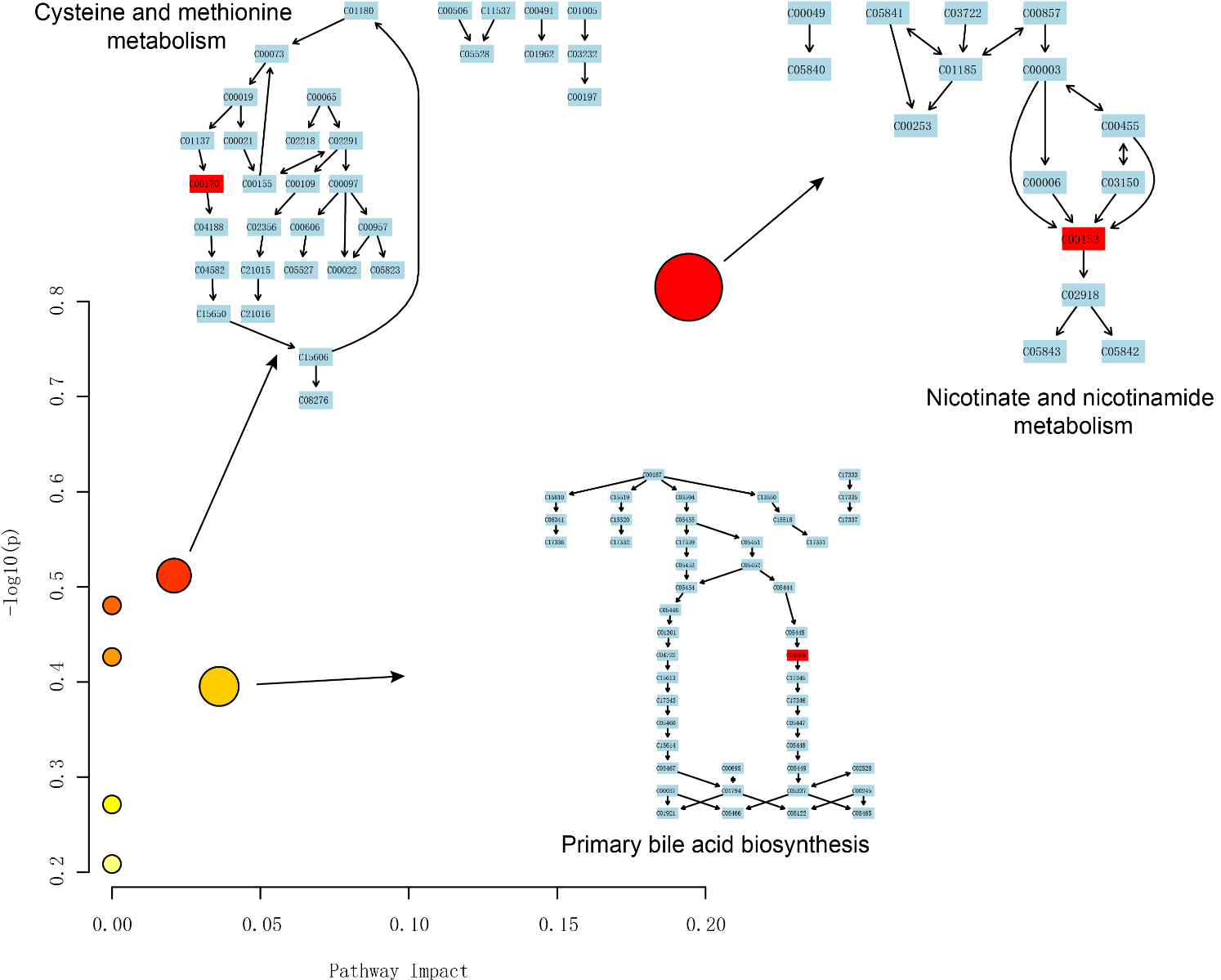
**

**Supplementary Figure 1.** Pathway analysis summary from MetaboAnalyst 5.0, using the significant metabolites changed in pre-PR vs pre-GR. The x-axis indicates the impact on the pathway whereas the y-axis indicates significant changes in a pathway. The top three contributing pathways nicotinate and nicotinamide metabolism, cysteine and methionine metabolism, and primary bile acid biosynthesis are shown.


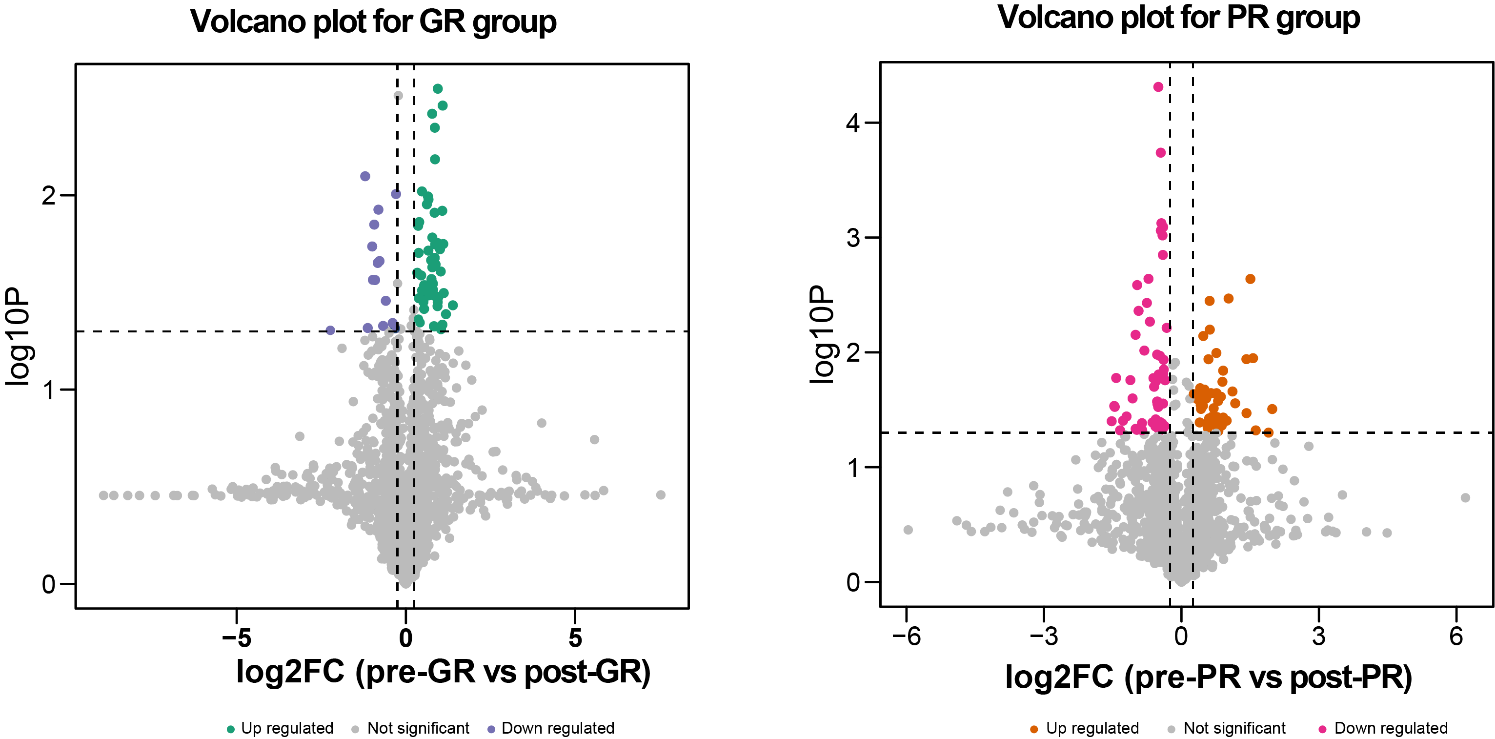


**Supplementary Figure 2.** Volcano plots for the differential metabolites between pre- and post-treatment in the GR group and the PR group, respectively. Significance is determined by using VIP of PLS-DA > 1, combined with |log2FC| ≥ 0.25 and the *Student's* T test P value < 0.05. *: the *Student's* T test P < 0.05; **: the *Student's* T test P < 0.01.


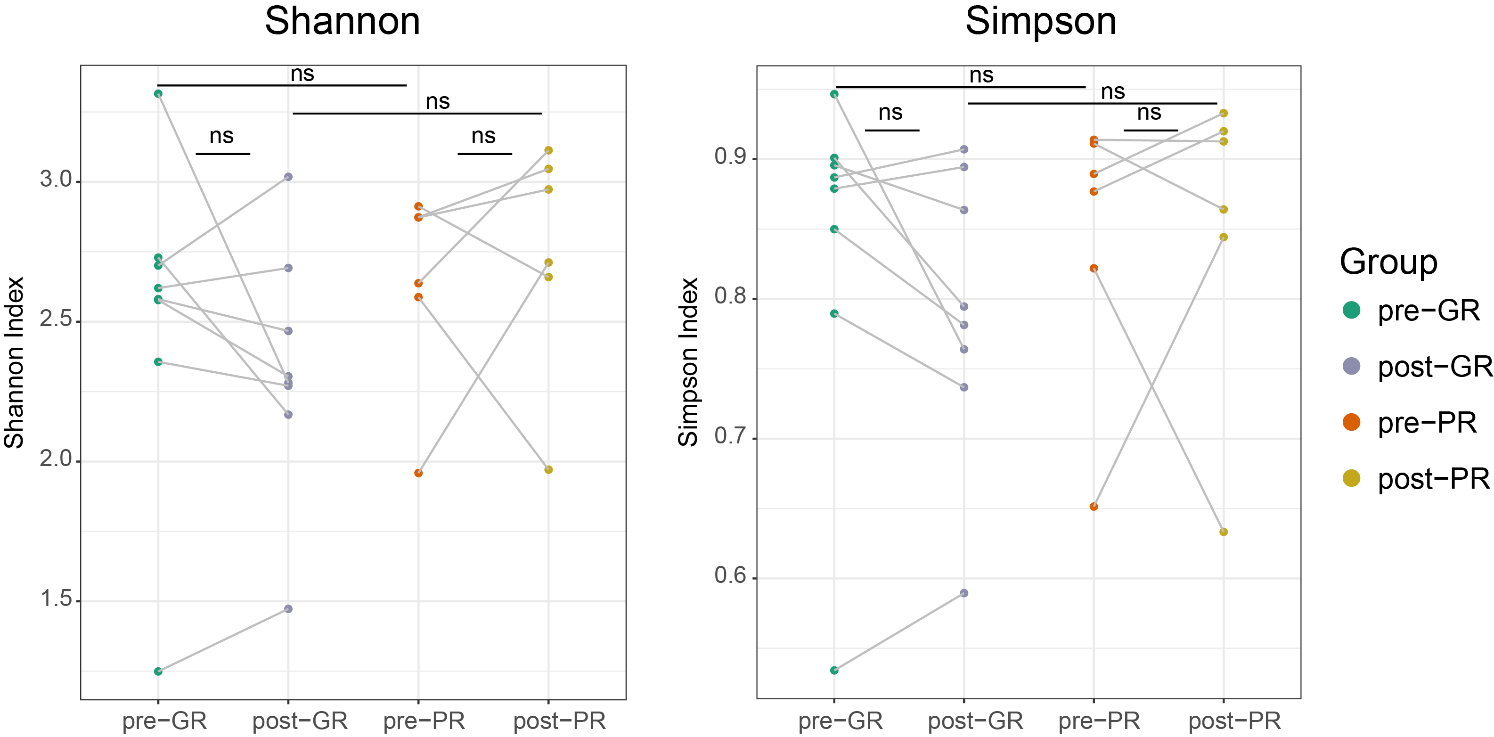


**Supplementary Figure 3.** Bacterial diversity analysis showed that no significant difference was observed in Shannon or Simpson index.


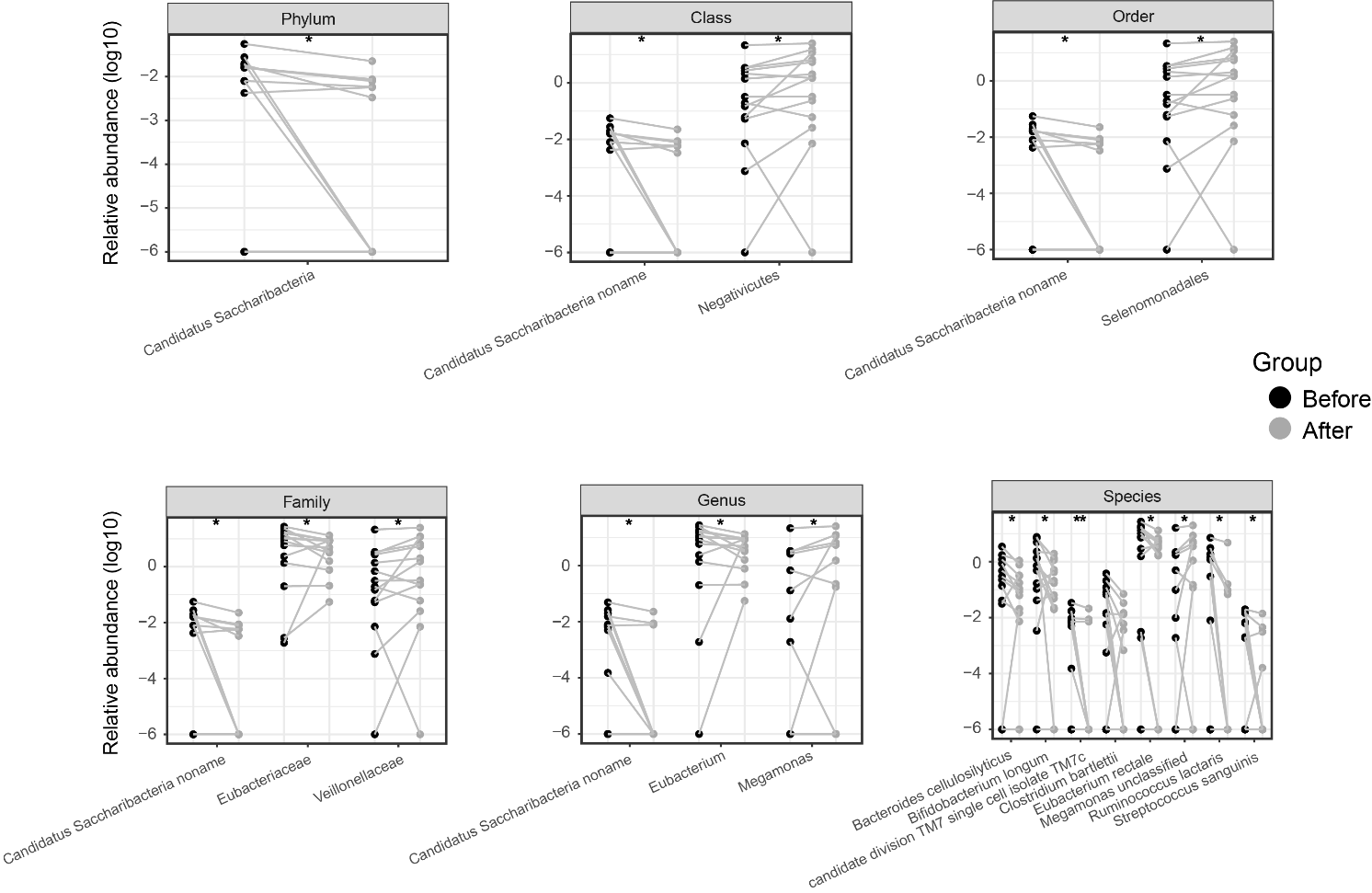


**Supplementary Figure 4.** Differential bacteria between pre- and post-treatment comparison. Significance is determined by the paired *Wilcoxon* test. *: the P < 0.05; **: the P < 0.01.


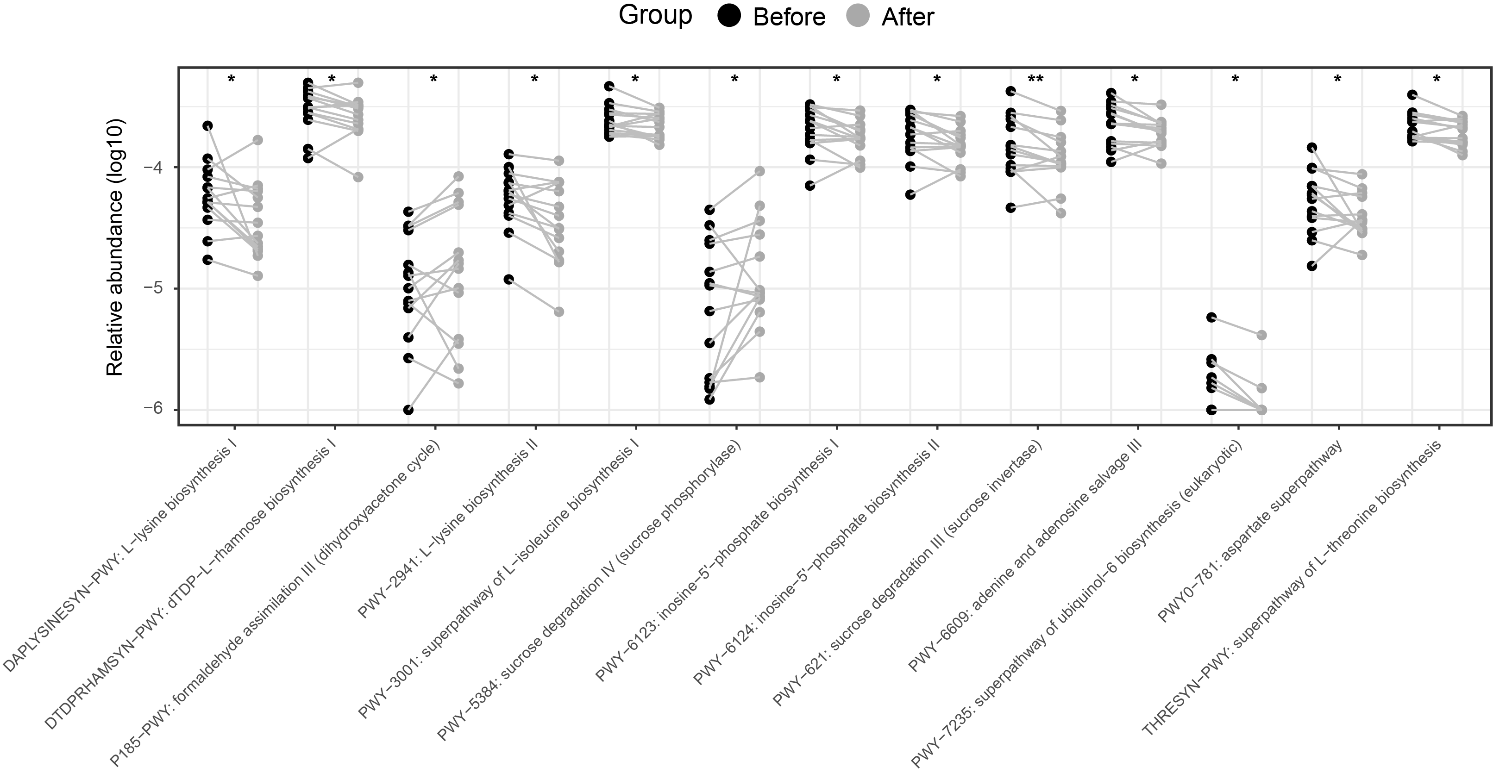


**Supplementary Figure 5.** Differential pathway between pre- and post-treatment comparison. Significance is determined by the paired *Wilcoxon* test. *: the P < 0.05; **: the P < 0.01.


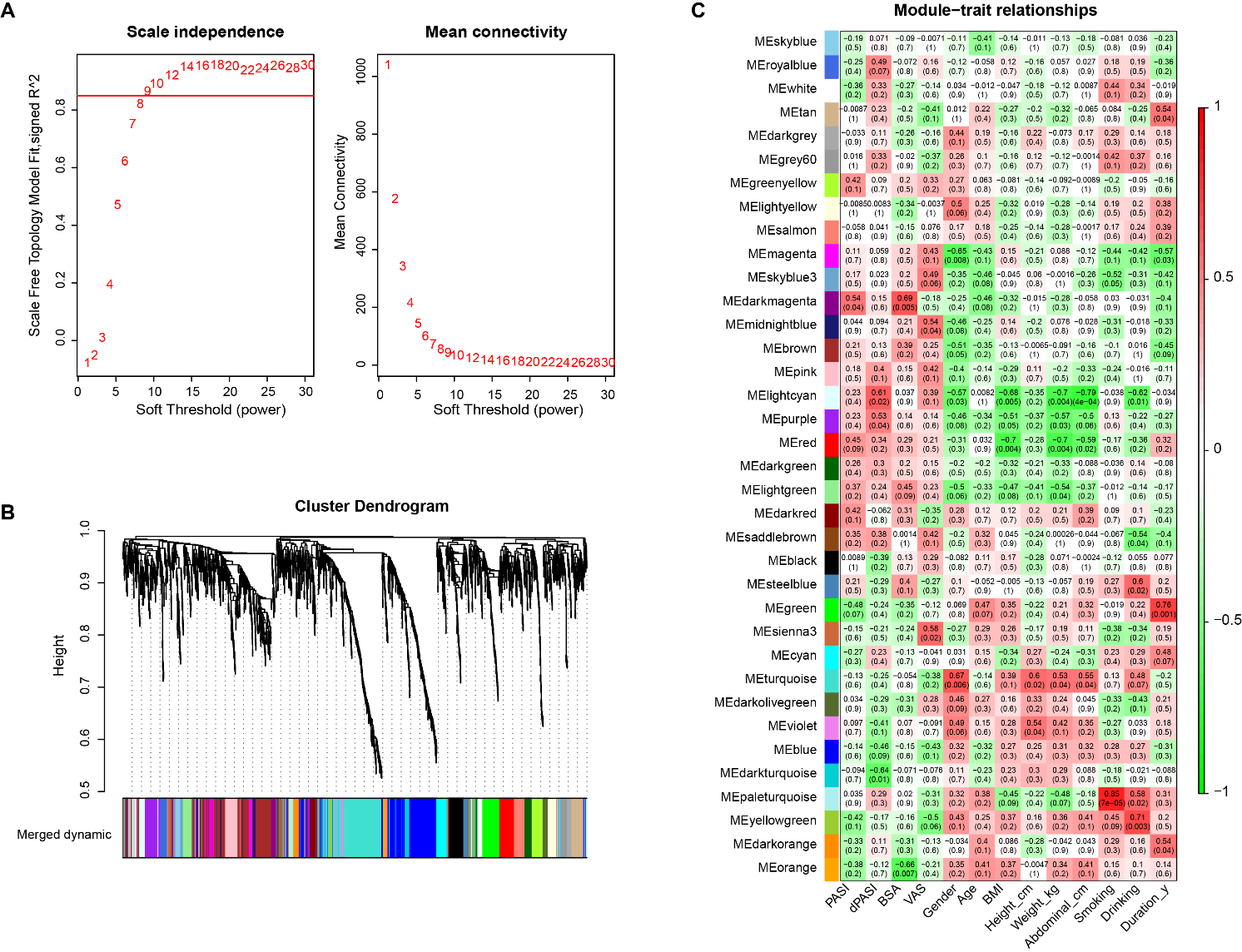


**Supplementary Figure 6.** WGCNA analysis for serum metabolome of the baseline samples. (A) Left: scale-free fit index (y axis) and the corresponding soft β value (x axis) in the network topology analysis; Right: the relationship between the β value (x axis) and the average node connectivity (y axis). (B) Cluster dendrogram of metabolites. (C) The relationships between metabolite clusters and traits. The Pearson correlation coefficient and the corresponding P value (in brackets) are showed in the cells.
